# An emerging potential of metabolomics in multiple sclerosis: a comprehensive overview

**DOI:** 10.1007/s00018-020-03733-2

**Published:** 2021-01-15

**Authors:** Insha Zahoor, Bin Rui, Junaid Khan, Indrani Datta, Shailendra Giri

**Affiliations:** 1 Department of Neurology, Henry Ford Hospital, Detroit, MI 48202 USA; 2 Department of Public Health Sciences, Henry Ford Health System, Detroit, MI 48202 USA; 3 Department of Neurology, Henry Ford Hospital, Education & Research Building, Room 4051, 2799 W Grand Blvd, Detroit, MI 48202 USA; 4 Department of Neurology, Henry Ford Hospital, Education & Research Building, Room 4023, 2799 W Grand Blvd, Detroit, MI 48202 USA

**Keywords:** Biological matrices, Biomarkers, Diagnosis, EAE, Metabolites, MS, Therapeutics

## Abstract

Multiple sclerosis (MS) is an inflammatory demyelinating disease of the nervous system that primarily affects young adults. Although the exact etiology of the disease remains obscure, it is clear that alterations in the metabolome contribute to this process. As such, defining a reliable and disease-specific metabolome has tremendous potential as a diagnostic and therapeutic strategy for MS. Here, we provide an overview of studies aimed at identifying the role of metabolomics in MS. These offer new insights into disease pathophysiology and the contributions of metabolic pathways to this process, identify unique markers indicative of treatment responses, and demonstrate the therapeutic effects of drug-like metabolites in cellular and animal models of MS. By and large, the commonly perturbed pathways in MS and its preclinical model include lipid metabolism involving alpha-linoleic acid pathway, nucleotide metabolism, amino acid metabolism, tricarboxylic acid cycle, d-ornithine and d-arginine pathways with collective role in signaling and energy supply. The metabolomics studies suggest that metabolic profiling of MS patient samples may uncover biomarkers that will advance our understanding of disease pathogenesis and progression, reduce delays and mistakes in diagnosis, monitor the course of disease, and detect better drug targets, all of which will improve early therapeutic interventions and improve evaluation of response to these treatments.

## Introduction

Multiple sclerosis (MS) is a progressive demyelinating disease of the central nervous system (CNS) that is caused by an inappropriate immune response against bodily components, leading to neurodegeneration that is accompanied by a continuum of clinical manifestations [1]. It is considered a significant cause of severe, irreversible neurological morbidity in young adults and has significant psychological and financial implications on patients and their families [2]. There is mounting evidence to suggest environmental (non-infectious or infectious) and genetic factors alter MS risk, with the exact trigger behind disease development still unknown [3]. Consequently, this complicates the accurate diagnosis, prognosis, and management of the disease [4]. MS pathogenesis is characterized by an aggressive immune response against the myelin sheath covering nerve fibers, which leads to excess inflammation in the body resulting in neuro-synaptic injury and destruction of myelin, white matter, neurons, axons, and blood vessels [5–8]. Both innate and adaptive arms of the immune system are involved in disease pathogenesis with both myeloid cells and lymphocytes playing critical role in mediating disease process [9–11]. The disease course is largely influenced by the immune response triggering the symptoms. The classical course of disease includes clinically isolated syndrome (CIS), relapsing–remitting (RR), secondary progressive (SP), and primary progressive (PM) [12]. CIS is considered as the first episode of neurologic symptoms due to immune activity that lasts for a 24-h period and may or may not develop into full bloom MS. RRMS is the most common disease course diagnosed in about 85% of the patients. It is characterized by episodes of relapse followed by recovery or remission; however, it may also change into progressive worsening where it develops as SPMS. While as, PPMS develops as progressive course from disease onset when first symptoms appear. This implies that MS disease course is very unique in a way that each stage has its own pathological and neurological implications that need to be dealt with specific diagnostic and therapeutic approach.

Experimental autoimmune encephalomyelitis (EAE) is the animal model that is widely used to study the immunopathological and neuropathological mechanisms in MS [13, 14]. In this model, mice develop an immune response against myelin antigens, which triggers inflammation followed by damage to myelin that eventually causes ascending paralysis, with key pathological features similar to MS. The findings from studies of EAE have made it clear that during the course of MS, heightened immune activation disrupts the blood–brain barrier through which activated immune cells gain access into the CNS and cause damage to oligodendrocytes, axons, and neurons, ultimately resulting in demyelination [15, 16]. Given the consequences of this immune response, there is currently research underway to develop promising therapeutic options that restore the cellular structures and functions damaged by heightened immune activation. In particular, there is a focus on restoring the balance between physiological and pathological inflammation by promoting endogenous process of inflammation resolution mediated by resolution mediators [17].

It is likely that pathological events in MS give rise to an altered metabolome. Studies strongly indicate that metabolic dysregulation in MS has a profound impact on the pathophysiology of disease, highlighting the importance of identifying alterations in metabolic by-products of biological processes to pinpoint the relevant pathways that contribute to disease progression [18–20]. Metabolic disruption creates changes in the metabolome (i.e., altered levels of metabolites or metabolic signatures derived from metabolic pathways), which can be monitored in biological samples from humans and preclinical disease models. Metabolic disruption is often misdiagnosed in individuals with MS, given its highly variable clinical and imaging presentation, several features of which are shared with other demyelinating diseases similar to MS (e.g., neuromyelitis optica spectrum disorder (NMOSD), Guillain–Barré Syndrome (GBS), and acute disseminated encephalomyelitis (ADEM)) [21–23]. For example, the conventional serological marker to separate neuromyelitis optica (NMO) from MS is the presence of aquaporin-4 (AQP4) antibodies in NMO patients. However, the detection of AQP4 antibodies is neither efficient nor sensitive enough to provide an absolute diagnosis and prognosis of NMO, and reliance on this marker alone to distinguish between the two diseases can increase the chances of misdiagnosis. Since the treatment modalities for non-MS diseases and MS are quite different, misdiagnosis can lead to the wrong treatment plan. As such, there is a need to identify methods that can provide a differential diagnosis between diseases that mimic MS. The metabolite profiling would identify distinct metabolites that are present in MS patients versus non-MS patients and could even be potential biomarkers that would help improve diagnosis of MS. Thus, while promising, the metabolomics approach needs further refinement to generate meaningful data that can be translated to the clinic, and the current focus should be on using multiple platforms that would generate more stage-specific reproducible data with the ultimate goal of improving disease management in MS patients.

Despite the fact that there are several drugs that are approved by the US Food and Drug Administration (FDA) to suppress inflammation and reduce the rate of relapse in MS patients, these are only partially effective and are often accompanied by substantial adverse effects [24]. In addition, we are currently limited in our ability to differentiate between progressive and non-progressive phases of MS, but doing so could enable stage-specific therapeutic intervention, which would aid in treating each course according to severity of the immune response and thereby modify further progression that would limit disease worsening to some extent. Therefore, identifying biomarkers that are specific to MS could provide a more accurate and timely diagnosis of the disease and enable early therapeutic intervention to halt disease progression in its initial stages, particularly in the case of the severely disabling progressive form of the disease.

In this review, we present a comprehensive analysis of the metabolomics studies that have been carried out in the context of MS. As mentioned above, identifying differences in the metabolome of MS patients versus healthy individuals could uncover biomarkers, leading to improved diagnostic capabilities. In addition, this could help decipher the primary metabolic pathway(s) that are altered in disease and provide potential targets for more accurate and effective therapeutic options. Here, we provide a general outlook of the metabolomics and technical advancements that have thus far enhanced the quality, accuracy, and reproducibility of metabolome studies, and which lay the groundwork for future studies in this area.

## Metabolomics: an overview

Metabolomics is a continuously growing, data-driven, high-throughput field that aims to identify and measure the concentrations of metabolites, which are low-molecular-weight small molecules (< 1 kDa) and are the offshoots of metabolic pathways connecting various biological processes [25, 26]. By illuminating specific signature elements of phenotypes that differentiate between health and disease, the metabolome has become a cornerstone for understanding the difference between physiological and pathological processes [27, 28]. Metabolites can be measured in a variety of biological samples and provide a global view of the functional status of the organism and a direct glimpse of abnormal or pathologic phenotypes [29, 30]. There are two approaches of measuring metabolites, untargeted-discovery-global and targeted-validation-tandem [31, 32]. Untargeted approach is used to generate hypothesis that enables global detection of all metabolites (> 1000) in a biological sample, giving a broader picture of whole metabolome and linked phenotypes. It is generally regarded as hypothesis-generating discovery-based model for relative quantification of metabolites using non-targeted profiling, fingerprinting, and footprinting strategies [33]. However, the unavailability of standards for some metabolites leaves them undetected. On contrary, targeted approach is driven by testing of a prior hypothesis enabling absolute quantification of specific but limited metabolites upto 20. It is generally used for validation of metabolites discovered during untargeted analysis using strategies such as target analysis and diagnostic analysis [31]. It is recommended to employ both approaches for a particular metabolomics experiment to achieve precise identification and complete quantification.

**Fig. 1:**
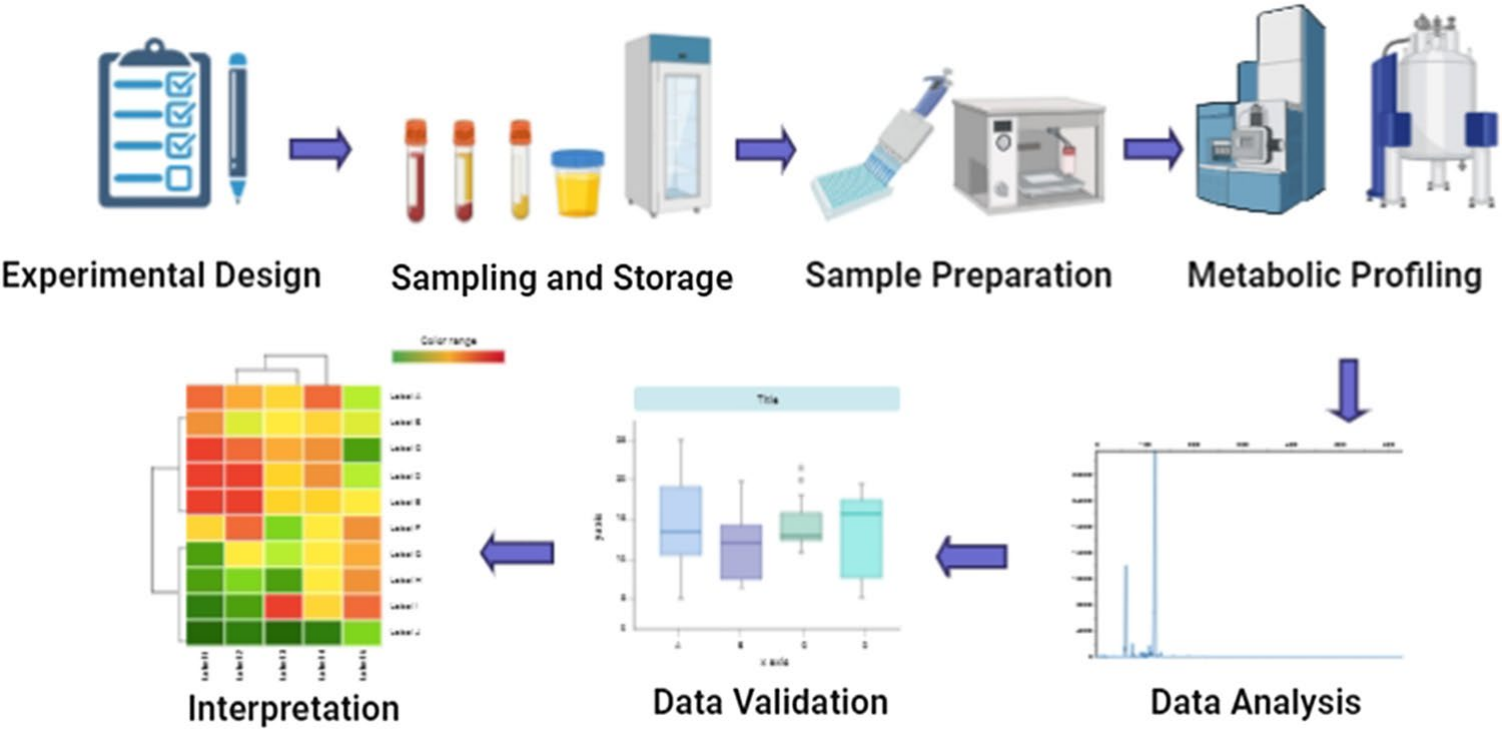
Schematic showing general workflow for a classic metabolomics study. It includes several steps, such as experimental design, sample preparation and storage, profiling, data analysis, validation, and interpretation (Created with BioRender.com (2020); https://app.biorender.com)

As a technique, metabolomics has become popular for studying disease pathogenesis as it often involves comprehensive characterization of biomarker targets and provides an overall snapshot of the function of biological processes vis a vis health of an organism [34]. It has been significant for advancing biomarker discovery for disease diagnosis, pathogenesis, and treatment responses [35], which continue to further advance as metabolomics is integrated with data from other omics techniques, such as genomics, transcriptomics, and proteomics. The general steps involved in a typical metabolomics experiment include: experimental setup, sample collection and storage, sample preparation, metabolite profiling and data analysis, validation, and interpretation. The schematic showing flow chart of basic steps of a metabolomics study is shown in Fig. 1.

## The utility of metabolomics in multiple sclerosis

**Fig. 2:**
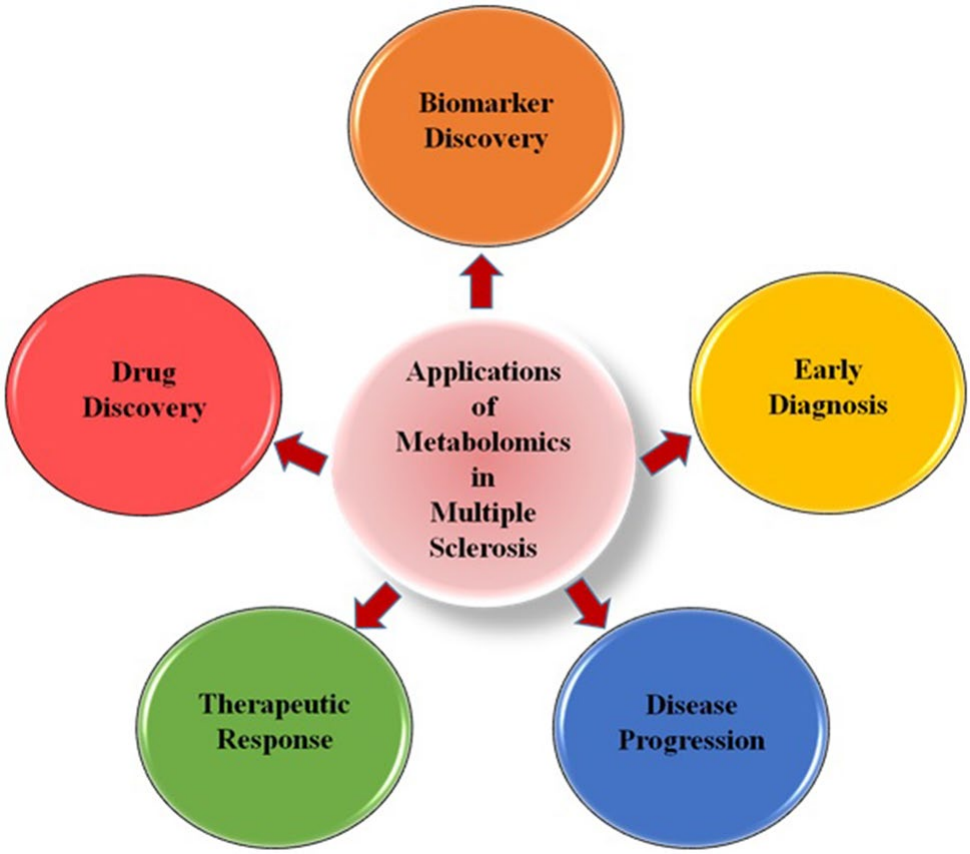
Basic applications of metabolomics that contribute to multiple aspects of understanding, detecting, and treating multiple sclerosis

Metabolomics studies have been used extensively to further understand metabolic dysregulation in MS due to the ability to profile the complete suite of metabolites with nuclear magnetic resonance (NMR), gas chromatography–mass spectrometry (GC–MS), liquid chromatography–mass spectrometry (LC–MS), and capillary electrophoresis–mass spectrometry (CE–MS) platforms on samples ranging from plasma, serum, urine, cerebrospinal fluid (CSF), and brain tissue from MS patients and the preclinical animal model EAE [18, 19, 36–42]. While it is common to use multiple platforms and biological sample types for metabolomics studies, the majority of initial studies utilized a single platform for metabolite profiling, which could be the reason only limited alterations in metabolic profiles were observed in MS patients. However, over the past few decades, there has been tremendous progress that has identified specific metabolic profiles in patient-derived and EAE samples. These studies have opened up new tools for discriminating MS from healthy subjects and also other MS-related diseases, as well as advanced the discovery of novel therapeutics to treat multiple stages of the disease (Fig. 2). Although these studies have uncovered new avenues of investigation, given that this is a relatively new field, the conclusions drawn from these studies are variable due to discrepancies in the methods employed for sample collection and preparation, as well as those used for data analysis. In the next section, the utility of metabolomics in MS and EAE is discussed, based on the outcome from some of the major studies, their sample size, nature of the biological matrix used for metabolite profiling, and metabolomics approach and platform utilized. Despite recent progress and the promising advances gained from metabolomics studies, there are still unanswered questions that need to be addressed for the benefits of this field to be fully utilized and improve our understanding of the pathogenesis of disease, which will allow us to make advances in the diagnosis and treatment of MS. As such, a thorough review of these studies will help identify the measures that provide the most robust data from which we can draw useful conclusions with clinical relevance, which we provide in the sections that follow.

## Overview of metabolomics studies in MS patient samples

### CSF-based metabolic profiling

There are a number of metabolomics studies using samples from MS patients that have shown various degrees of abnormalities in the metabolome that are derived from different biological samples. Most of the initial studies have been conducted on CSF, since it serves as the interface between the blood and brain tissue and provides a direct picture of MS pathology and brain activity at the initial site of the autoimmune attack. This makes analysis of the CSF an important tool for disease diagnosis and prognosis [43, 44]. To this end, some major studies assessing the metabolome in CSF samples from MS patients are described below.

One of the early metabolomics studies in MS, which dates back to the 1990s, performed metabolic profiling in an untargeted approach using proton (1H)-NMR spectroscopy of the CSF from 30 progressive MS patients and 27 controls with other neurological diseases [45]. This study revealed significantly higher levels of acetate and lower levels of formate in the CSF of MS patients compared to controls, reflecting perturbed energy metabolism. No difference was observed in the profiles between patients with early and late-stage MS. Another study assessed the CSF from 19 MS patients and 17 controls using proton magnetic resonance spectroscopy (1H-MRS) and demonstrated that the CSF from MS patients had higher levels of lactate and fructose, and lower levels of creatinine and phenylalanine relative to controls [46]. However, there was no difference in the metabolic profile between those patients diagnosed with the RRMS or PPMS form of the disease. Similarly, Simone et al. utilized MRS with magnetic resonance imaging (MRI) to also show increased levels of lactate in the CSF of patients with active lesions and decreased levels of formate in patients with or without active lesions, reflecting abnormality in glucose and energy metabolism pathways [47]. However, these findings were inconsistent with a separate study showing decreased lactate and glutamine levels in the CSF of MS patients compared to controls [48]. Therefore, findings from these metabolomics studies need validation in large cohort of patient and control samples before making any conclusions.

In the first investigation that utilized MRS on a homogeneous cohort of patients with CIS, unique metabolic profiles were reported in CSF samples derived from 21 patients with active disease versus CSF samples from 12 patients with inactive inflammatory brain plaques, hinting towards selective metabolic dysregulation during active and inactive phases of disease [39]. Out of 27 detected metabolites, enhanced levels of beta-hydroxyisobutyrate (BHIB) were observed in patients with active plaques compared to those with inactive plaques. In addition, a significant correlation was found between lactate levels and the number of plaques in the brain. Altogether, the findings from this CSF metabolome analysis suggest a correlation between plaque activity and defects in organic acid metabolism, indicating metabolic dysfunction plays a role in governing disease severity.

In a pilot study to examine the association between energy metabolism and disease progression in MS, archived CSF samples from 85 MS patients (31 RRMS and 54 SPMS) and 18 healthy controls were analyzed using GC–MS [49]. This revealed a significant increase in the levels of sorbitol, fructose, and lactate in the samples from patients with SPMS, and a slight increase in these levels in patients with RRMS, compared to controls. The elevated levels of sorbitol and fructose had a strong positive correlation with neurologic disability in MS patients. In addition, they also observed a correlation between increased extra-mitochondrial glucose metabolism and disease progression, implicating mitochondrial dysfunction in MS pathogenesis. This study was pivotal in highlighting the prospect of targeting processes that regulate energy metabolism for therapeutic intervention in MS. Similarly, another study based on 1H-NMR spectroscopy and multivariate pattern recognition analysis techniques identified a unique metabolic profile in the CSF and serum of MS patients compared to patients with idiopathic intracranial hypertension (IIH), cerebrovascular disease (CVD), and other mixed neurological diseases [50]. In this study, MS patients showed significantly reduced citric acid cycle intermediates (oxaloacetate and citrate). Taken together, these studies suggest abnormalities in the metabolites derived from glucose metabolism.

In one of the first significant studies on CSF samples from 9 MS patients and 9 non-MS patients using an untargeted metabolipidomics approach with the LC–MS/ultra high-pressure liquid chromatography (UHPLC)-MS platform, a clear separation of multiple altered metabolites and lipids was observed between the two sample groups [51]. Targeted pathway analysis further showed higher levels of the oxidative stress marker 8-iso-prostaglandin F2α in MS patients as well as elevated levels of autoantibodies against lipoxidized proteins, suggesting a pathogenic autoimmune response against lipid peroxidation-modified proteins contributed to disease progression.

Most of the initial NMR-based metabolomics studies have used low-field strength NMR spectrometers, which have sensitivity limitations in detecting metabolites. In contrast, Reinke et al. used high-field strength 1H-NMR spectroscopy (800 MHz) and multivariate hierarchal cluster data analysis with principal component analysis (PCA) for metabolomic profiling of CSF from 15 MS and 17 non-MS patients [41]. They detected a total of 15 metabolites with a clear separation of profiles between the 2 clinical groups. This included alterations in energy and phospholipid metabolism with increased levels of choline, myo-inositol, and threonate, and decreased levels of 3-hydroxybutyrate, citrate, phenylalanine, 2-hydroxyisovalerate, and mannose in MS patients, which were consistent with other previously published studies [46, 47, 50]. Collectively, those studies which have demonstrated higher levels of lactate and reduced levels of citric acid cycle intermediates suggest glycolysis is enhanced and mitochondrial function is decreased in MS.

Multiple studies have analyzed the CSF metabolome in MS patients, and a recent study utilized integrated metabolomics by applying a combination of matrix-assisted laser desorption ionization (MALDI)-time of flight (TOF)-MS untargeted lipidomics and targeted LC–MS/MS analysis on CSF samples from 13 patients with RRMS and 12 controls with other neurological diseases (non-MS). This identified 10 metabolites that were clearly distinguished between the two groups, including elevated levels of multiple lipids in MS patients. Among elevated lipids, the most significant alterations were in phospholipids, some of which showed correlation with disease activity, disease duration, and disability score in patients [52]. One of the major studies to perform comparative metabolomics analysis between MS and NMOSD employed 1H-NMR with univariate and multivariate analyses to characterize the metabolic profile in the CSF from 50 MS, 57 NMOSD, and 17 control samples [53]. A total of 8 metabolites were found to be altered among subjects, including increased levels of 2-hydroxybutyrate, acetone, formate, and pyroglutamate, and decreased levels of acetate and glucose in both MS and NMOSD samples. Citrate was lower in MS patients, and lactate was elevated only in NMOSD patients. The altered metabolite levels clearly suggest abnormalities in energy metabolism and fatty acid biosynthesis. Further, levels of isoleucine and valine were reduced in MS patients undergoing a relapse, relative to those in a state of remission. Thus, this study is significant in demonstrating the potential of metabolic changes that differentiate MS from NMOSD, as well as those that isolate differences in conditions of an immune attack relative to a state of remission.

To discriminate between different stages of MS, an integrated approach was used by combining MRI findings with changes observed in the levels of proteins and metabolites from the CSF of 30 RRMS, 16 SPMS, and 10 control subjects with other non-inflammatory neurological diseases (non-MS) [54]. Using enzyme-linked immunosorbent assay (ELISA), LC–MS, and clinical data from MRI, 11 variables were assessed, which included the 2 metabolites 20β-dihydrocortisol (20β-DHF) and indolepyruvate, 3 MRI variables, and 6 proteins, including galectin-9, monocyte chemoattractant protein-1 (MCP-1), transforming growth factor alpha (TGF-α), tumor necrosis factor alpha (TNF-α), soluble CD40L (sCD40L), and platelet-derived growth factor AA (PDGF-AA). Distinct profiles were generated based on these variables and were used to monitor disease progression and disability by distinguishing MS subtypes from each other and controls. Overall, the elevated levels of myelin basic protein (MBP) and macrophage-derived chemokine (MDC) along with metabolites 20β-DHF and 5,6-dihydroxyprostaglandin F1a (5,6-DH-PGF_1_) in SPMS, were identified as potential biomarkers for assessing progression of disability. In a separate study using the same patient samples as just described (30 RRMS, 16 SPMS, and 10 non-MS), high-resolution MS (HRMS) of the CSF followed by partial least-squares discriminant analysis (PLS-DA) was used to assess changes in the metabolome that could discriminate MS subtypes and identify biomarkers for disease progression [55]. Collectively, the pathway with the greatest number of changes was tryptophan metabolism, as patients with SPMS showed elevated levels of kynurenate, kynurenine, indolacetate, 5-hydroxytryptophan, and N-acetylserotonin and reduced levels of 5-hydroxyindoleacetate, compared to patients with RRMS or controls. In addition, in patients with SPMS, pyrimidine metabolism was significantly altered compared to RRMS, as indicated by elevated levels of thymine, glutamine, and uridine and reduced levels of deoxyuridine. Overall, this study identified a strong correlation between altered levels of tryptophan and pyrimidine metabolites with disease duration, disability, pathology, and its progression, indicating the CSF metabolome is predictive of stage-specific disease pathology in MS.

To identify lipid profiles that were indicative of disease, Nogueras et al*.* applied a non-targeted lipidomic approach using LC–MS and GC–MS on CSF samples from 53 RRMS patients and 54 non-MS subjects. They identified MS-specific lipidomic signature comprising of 15 molecules from 5 lipid families. Targeted fatty acid analysis further showed clear differences in arachidic acid in samples from MS patients compared to non-MS patients, clearly highlighting the potential use of lipids as diagnostic biomarkers [56]. Likewise, Podlecka-Piętowska et al. have shown lower levels of hydrophilic and hydrophobic compounds in CSF from 19 MS patients as compared to 19 controls, by employing univariate and multivariate supervised orthogonal partial least square discriminant analysis (OPLS-DA) [57]. Specifically, the authors observed decreased levels of acetone, choline, urea, 1,3-dimethylurate, creatinine, isoleucine, myo-inositol, leucine, and 3-OH butyrate, which was in agreement with previous studies [50]. Overall, this study demonstrated disturbance in fatty acid synthesis and other metabolic pathways, which could possibly decrease the levels of acetyl-CoA. Therefore, the observed alteration in energy metabolism could impact the myelin repair and neurotransmission. Another recent study demonstrated the first application of the AbsoluteIDQ^®^ p400 kit (Biocrates Life Sciences AG) for targeted metabolomics using a high-resolution hybrid quadrupole-Orbitrap mass spectrometer to perform LC-HRMS and flow injection analysis (FIA)-HRMS on the CSF from 12 SPMS patients and 12 healthy controls [58]. This showed a significant increase in the levels of glycine, asymmetric dimethylarginine (ADMA), glycerophospholipid PC-O (34:0), and hexoses in SPMS patients compared to controls. They also showed superior reproducibility of LC-HRMS over FIA-HRMS. Likewise, Murgia et al*.* used a combination of NMR and MS/GC–MS/LC–MS to characterize the CSF and serum profiles from 22 RRMS and 12 PPMS patients [59]. This revealed distinct metabolite profiles, which included lipids, biogenic amines, and amino acids, with major metabolic pathways affected, including metabolism of glutathione, nitrogen, glutamine–glutamate, arginine–ornithine, and phenylalanine, and biosynthesis of tyrosine and tryptophan. Overall, the results of this study suggest that different levels of metabolites in the serum and CSF can differentiate between the relapsing and progressive phases of the disease. However, the limitations posed by non-inclusion of healthy control group and small sample size require validation of findings in larger cohorts.

So far, reports have revealed that the metabolite signatures that are most distinct in MS patients relative to controls are those obtained from analysis of the CSF, given its close association with the primary autoimmune attack in the CNS [60, 61]. However, obtaining samples of the CSF involve invasive procedure of lumbar puncture, making the acquisition of this material risky and unfeasible in certain situations [62]. In addition, analysis of the CSF does not hold significance for the early diagnosis of MS, which certainly potentiates the need to identify an alternative tissue type to profile during initial stages of the disease [63].

### Blood-based metabolic profiling

There has been an emphasis on finding alternative biological material that can be obtained in a manner that is less invasive, safe, and readily available, such as blood and urine [62]. Given this, the majority of recent metabolomics studies have been carried out in serum and plasma derived from the blood of MS patients to study peripheral pathological alterations due to easily accessibility, and the belief that whole blood represents a more informative and comprehensive picture of the metabolic status of the human body [19, 36, 61]. However, serum and plasma from the blood may be less sensitive in detecting metabolic changes occurring in the CNS due to filtration at the blood–brain barrier, which may limit its potential to reflect the true profile of the CNS, considerations which should be kept in mind when drawing comparisons with studies using the CSF [64]. Therefore, although there are certain limitations with the use of whole blood, the ease in obtaining samples and potential to detect biomarkers for diagnosis make it an attractive candidate.

One of the early comparative metabolomics studies in blood profiled lipids in serum from patients with MS, other neurological diseases (non-MS), or from healthy controls. LC–MS-based lipidomics revealed changes in lyso-glycerophosphatidylcholine (lysoPC) and glycerophosphatidylcholine (PC), with a significant decrease observed in the lysoPC/PC ratio in samples from MS patients, indicating an abnormality in phospholipid metabolism [65]. Altered energy and redox metabolism was reported in a study of 170 MS patients (113 RRMS, 43 SPMS, and 14 PPMS) in which serum was profiled using high-performance liquid chromatography (HPLC) [66]. In particular, MS patients showed increase in multiple metabolites, including uric acid, creatinine, hypoxanthine, xanthine, uridine, β-pseudouridine, malondialdehyde, nitrite, and nitrate, along with a decrease in ascorbic acid, compared to 163 healthy controls. 1H-NMR with a random forest classifier has also been used for metabolic profiling of serum obtained from 23 MS and 28 control subjects [67]. Atomic absorption showed lower levels of selenium in MS patients compared to controls. The main metabolite changes were increased levels of glucose and reduced levels of valine, which in turn disturbed the selenium levels in MS patients. However, there was no detailed information on other metabolites. Another study using GC–MS and a multivariate statistical analysis platform (PLS-DA) compared the plasma profiles of MS patients with healthy controls [68]. This identified multiple metabolites with altered expression between the two groups, including phosphate, fructose, myo-inositol, pyroglutamate, threonate, l-leucine, l-asparagine, l-ornithine, l-glutamine, and l-glutamate, and found that the asparagine and citrulline pathways were also altered in MS patients. These pathways were perhaps reflective of oxidative stress involved in neuronal damage observed in MS pathology. Notably, these findings were similar to those from a study that analyzed CSF samples using NMR approach [41]. Likewise, in another study, 1H-NMR spectroscopy and multivariate analysis (PLS-DA and OPLS-DA) were used to profile the plasma samples acquired from 73 therapy-free MS patients and 88 controls. This revealed that MS patients had decreased levels of glucose, 5-OH-tryptophan, and tryptophan, and increased levels of 3-OH-butyrate, acetoacetate, acetone, alanine, and choline relative to controls [69]. These metabolite changes were linked to changes in the tryptophan pathway and energy metabolism. Altogether, these studies imply utility of metabolomics to study disease-specific metabolite profile.

To detect the metabolites associated with relapse activity and disease disability in MS, untargeted metabolomics using UHPLC–MS was performed on serum samples from 2 cohorts, including a retrospective longitudinal cohort of 238 patients and 74 controls, and a prospective cohort of 61 patients and 41 controls [70]. PLS-DA and PCA analysis identified altered levels of multiple metabolites from lipids (e.g., glycerophospholipids, steroids, and oxidized fatty acids), hormones, and amino acids that were associated with more severe disability over time. These included hydrocortisone, glutamic acid, tryptophan, eicosapentaenoic acid, 13S-hydroxyoctadecadienoic acid, lysophosphatidylcholines, and lysophosphatidylethanolamines. Similarly, another study profiled 13 amino acids and 29 acylcarnitines in plasma acquired from RRMS patients during disease relapse using a targeted metabolomics approach with LC–MS/MS [71]. The general linear regression and random forest model generated by the data indicated a significant increase in the levels of glutamate coupled with a decrease in leucine–isoleucine and decenoylcarnitine in patient samples, suggesting acylcarnitines as novel biomarker candidates for MS.

Several studies have correlated changes in the metabolome with phenotypic data. A recent study used untargeted 2D GCxGC-TOF MS and targeted lipidomic and amino acid analysis on serum samples obtained from 12 non-Hispanic white non-smoking therapy-free male MS patients and 13 matched controls [18]. This study correlated metabolite changes with gene expression and genotype data, and random forest analysis showed 12 metabolites were significantly altered in MS patients compared to controls. In particular, 6 major metabolites were elevated in MS patients, including pyroglutamate, laurate, acylcarnitine C14:1, N-methylmaleimide, and 2 phosphatidylcholines (PC ae 40:5, PC ae42:5). Broadly, pathway analysis showed major perturbations in mitochondrial functioning, apoptosis, and energy metabolism. Although the expression analysis did not show an association of HLA-DRB1 with any metabolite, other HLA genes and non-MHC risk variants were associated with top 6 altered metabolites mentioned above. In addition, the major MS risk allele, HLA-DRB1 × 15:01, was associated with acylcarnitine C14:1. Overall, this study showed an association between changes in the levels of metabolites with gene expression and genomic data, which give an idea about pathways involved in MS pathology with potential of serving as novel targets for better diagnosis of MS and its treatment. In addition, another recent study used brain MRI and untargeted NMR with plasma samples from 28 RRMS and 18 controls to correlate peripheral metabolic disturbances with neuroanatomical characteristics [72]. This revealed significantly reduced concentrations of arginine, isoleucine, citrate, serine, phenylalanine, methionine, asparagine, histidine, and myo-inositol in the plasma of RRMS patients compared to controls. Notably, levels of arginine showed a positive correlation with brain atrophy and white matter lesions whereas levels of methionine were positively correlated with brain atrophy. Overall, this study is significant in revealing an association of plasma metabolites with structural changes in the brain of MS patients. Collectively, these studies demonstrate that phenotypic characteristics can be correlated with changes in the metabolome, yet given the small sample size used, the findings must be replicated with a larger sample size to strengthen these correlations.

One of the main objectives of metabolomics studies has been to identify mechanisms that can differentiate MS from other related diseases. Accordingly, Moussallieh et al. utilized 1H-NMR spectroscopy with multivariate pattern recognition analysis to identify metabolic markers across serum samples from 44 NMO and 47 MS patients, as well as 42 controls [73]. After eliminating outliers, PLS-DA models showed differences in the levels of multiple metabolites in samples from NMO and MS patients. Specifically, compared to control samples, those from MS patients showed an increase in scyllo-inositol and glutamine, whereas samples from NMO patients showed elevated levels of acetate, glutamate, lactate, and lysine. Of these altered metabolites, differences in scyllo-inositol and acetate could discriminate between MS and NMO with high sensitivity and specificity. However, repeating this study is necessary to ensure the reproducibility of these data, as the MS patient group included 39 patients that were treated with natalizumab and 3 that were treated with interferon, which could interfere with the metabolomic profiles observed. In line with this study, distinct plasma metabolite profiles that were capable of differentiating RRMS from AQP4-antibody (Ab) NMOSD and MOG-Ab diseases, were identified through the use of an NMR platform with OPLS-DA statistical modeling [74]. In this study, elevated levels of large, low-density lipoproteins and decreased levels of scyllo-inositol and small high-density lipoproteins were observed in samples from patients with NMOSD compared to those with RRMS and MOG-Ab disease. Further, RRMS patient samples showed an increase in the levels of histidine and glucose and decreased levels of lactate, alanine, and large high-density lipoproteins. In contrast, MOG-Ab patient samples had elevated levels of formate and leucine, along with low levels of myo-inositol. Collectively, these studies are significant in demonstrating distinct metabolomic profiles exist for these diseases, which suggests the importance of metabolomics in identifying disease-specific signatures, which can be implemented as diagnostic tools when clinical assessments fail to provide a differential diagnosis.

Another significant objective of metabolomics studies in MS is to develop tools that enable the progression and severity of the disease to be monitored, based on changes in metabolite levels. In a large targeted metabolomics study, HPLC analysis was used to assess serum samples from 518 patients with MS and 167 healthy controls. This identified 9 compounds, including hypoxanthine, xanthine, uric acid, inosine, uracil, β-pseudouridine, uridine, creatinine, and lactate which were significantly different between patients and controls [75]. The altered metabolites were linked to mitochondrial dysfunction and were strongly associated with a progressive disease course, high disability score, and neuroanatomical alterations. This study was quite significant in revealing link between impaired energy metabolism and neuroimaging observations in form of lesions.

In another study, UHPLC and GC–MS were used to profile the kynurenine pathway, which is involved in tryptophan metabolism, using serum samples from cohorts of patients with RRMS, SPMS, PPMS, and controls [76]. Metabolomics data analysis revealed abnormalities in the kynurenine pathway associated with disease progression and disability score, which could distinguish between the different MS subtypes, with significantly altered levels of kynurenic acid and quinolinic acid observed and elevated kynurenine/tryptophan ratio in patients. This was consistent with findings from a study that used LC/GC–MS in a global untargeted metabolomics approach to explore the association of tryptophan metabolism by the gut microbiome and the kynurenine pathway with MS risk and disease course in 66 pediatric and CIS cases and 66 pediatric controls, with targeted tryptophan metabolism in a discovery group (82 pediatric cases, 50 pediatric controls) and a validation group (92 pediatric cases, 50 pediatric controls), and functional microbiome analysis in 17 pediatric cases [77]. This showed a correlation between a higher relative abundance of tryptophan and indole lactate with lower MS risk and lower disability, and a higher relative abundance of kynurenine with a higher relapse rate in pediatric cases. Overall, these studies showed a correlation in altered tryptophan metabolism with the risk, activity, and severity of MS.

In another significant study, various courses of MS were differentiated by assessing metabolite profiles from serum samples obtained from patients with RRMS, SPMS, and PPMS, and patients with other neurodegenerative conditions (non-MS, negative control), and matched healthy controls using NMR spectroscopy with PLS-DA analysis [78]. For each MS cohort, different profiles for each group were observed compared to controls. However, the distinction was more significant and precise between samples from RRMS and SPMS patients, as shown by a type II biomarker. Although the results of this study suggested metabolic changes play a role in driving the transition between the relapsing stage of MS to the progressive stage, the lack of specificity with regard to changing metabolite levels lowers the overall confidence in the described outcomes.

In a recent study, MS-associated stage-specific metabolite profiles were assessed in plasma samples derived from different cohorts that comprised of 33 PPMS, 10 RRMS, 63 healthy controls, and 40 samples from patients with Parkinson’s disease [79]. Using LC–MS and PLS-DA in an untargeted high-resolution metabolomics approach (HRMS), stage-specific metabolites were observed in a cohort of PPMS patients over the course of 2 years follow-up. PLS-DA analysis revealed a panel of 20 discriminatory metabolites that were deregulated in patients with PPMS, mainly linked to alterations in glycerophospholipid and linoleic acid pathways, with consistently reduced levels of lysoPC during 2 years of disease course. These findings demonstrated the potential for changes in the levels of phosphatidylcholine species to serve as biomarkers that can be helpful in accurate diagnosis of MS in early stage, disease sub-type classification, and monitor disease course over time. Altogether, results from blood-based metabolite profiling have been crucial in enhancing our knowledge of the altered metabolome in MS.

### Urine-based metabolic profiling

Similar to blood, urine is also a non-invasive source of metabolites that is quick, safe, and easy to obtain [80]. Since it is not under homeostatic control, it may provide a better reflection of the early metabolic changes that occur in MS relative to plasma [81]. However, there are only a handful of studies that have used urine as a material to identify differences in the metabolome of MS patients. Hence, there is untapped potential in urinary metabolite profiling that may provide important warning signatures for biomarker discovery in MS. In an initial study, metabolic profiling of urine samples was performed using 1H-NMR spectroscopy combined with pattern recognition techniques to compare samples from 10 MS patients with 11 healthy controls and 20 patients with other neurological diseases (non-MS) [82]. Through PCA and PLS-DA analysis, distinct metabolic profiles of each group were found, with preliminary findings showing differences in aspartic acid, inositol, and taurine, which was in agreement with findings from serum, CSF or brain tissue of MS patients and EAE [83–86]. Altogether, this study revealed the potential to use urine samples to track metabolic dysfunction in MS.

To determine the ability of metabolic profiles to be detected in urine samples that were able to differentiate MS patient samples from those of healthy controls and NMO patients, Gebregiworgis and colleagues applied NMR, univariate and multivariate statistics, and one-way analysis of variance (ANOVA) to study the changes in urinary metabolites [87]. Using 38 samples, including 8 MS, 9 NMO, and 7 HS, the authors observed a unique metabolic profile for samples from MS patients compared to samples from NMO patients and the control group, with a total of 27 differentially altered metabolites. The specific metabolites that differed between samples from MS patients and those from NMO patients included creatinine, 3-hydroxybutyrate, 3-hydroxyisovalerate, and methylmalonate. Altered metabolites in urine from MS patients were related to energy and fatty acid metabolism, mitochondrial activity, and the gut microbiota. Thus, this study demonstrated the potential for urinary metabolites to serve as prospective biomarkers that can identify a differential diagnosis of MS. However, given the low sample size of the study, the findings need to be replicated in a larger patient cohort prior to any advances being made on these conclusions.

Using a targeted approach, Gaetani and colleagues measured tryptophan metabolites by HPLC–MS/MS in the urine of 47 RRMS patients and 43 healthy controls [88]. The authors observed differentially expressed urinary tryptophan metabolites in patients with a recent relapse, with lower kynurenine/anthranilate ratio in relapsing patients compared to stable patients. RRMS patients showed lower levels of kynurenine and kynurenine/tryptophan ratio than controls, with kynurenine/tryptophan ratio negatively correlated with disability score. Relapsing patients had significantly higher levels of indole-3-propionic acid than stable patients, which showed positive correlation with disability score. Altogether, these findings suggest association of altered tryptophan metabolism with RRMS, in which reduced tryptophan metabolism through kynurenine pathway results in lower levels of kynurenine associated with disease severity. Taken together, the above studies highlight the utility of non-invasive urinary metabolomics in finding a specific novel urinary signature by identifying impaired metabolic pathways that would serve as potential drug targets for MS.

### Other biological matrices used in metabolic profiling of MS

The most appropriate non-invasive approach to follow disease progression in MS remains MRI, which can monitor lesion patterns in the brain. However, this technique comes with certain limitations of low sensitivity and limited specificity, which creates chances of misdiagnosis [89]. To improve studies based on this technique, advanced computational metabolomics involving multivariate statistical analysis models, such as PLS-DA and OPLS, have been applied to in vivo neuroimaging study that used 1H-MRS to identify metabolic changes in frontal lobe white matter of 27 patients with RRMS and 14 controls [90]. This demonstrated that RRMS patients had a unique metabolic profile compared to controls, but this was not precisely correlated with disease diagnosis. However, associations between clinical features and the frontal lobe metabolome could be identified by combining metabolomics with human brain spectroscopy. *N*-acetyl aspartate (NAA), choline, myo-inositol, and glutamine/glutamate ratio showed negative correlation with global neurological impairment, lower levels of NAA, choline, and glutamine/glutamate along with increase in scyllo-inositol were correlated with loss of verbal memory, possibly reflecting decline in cognition. The authors correlated lower levels of metabolites to change in creatine and phosphocreatine (Cr + PCr). Altogether, this study provided a unique experimental design for metabolome-based brain imaging in MS, which could be used with a large sample size to confirm the ability to track disease progression and treatment responses in individual patients with this approach.

An additional sample type that may hold promise for use to detect relevant biomarkers of MS is that of easily accessible lacrimal fluid in tears. In a recent study using integrated lipidomics with a metabolomics approach, LC–MS/MS was used to compare the diagnostic potential of lacrimal fluid from the tears of 12 MS patients and 21 controls, relative to the sera from 12 MS patients and 10 controls [91]. This revealed abnormalities in 30 phospholipids, with significantly lower levels of sphingomyelins in the tears of MS patients compared to controls. Taken together, these data revealed the diagnostic potential of phospholipids, amino acids, and acylcarnitines in MS. Taken together, the above-discussed studies on metabolite profiling in different biological fluids from MS patients underscore the utility of metabolomics as a powerful tool for evaluating metabolic differences in form of a specific MS-related biomarker that may facilitate distinguishing MS from non-MS overlapping diseases and monitoring disease progression by discriminating different disease stages.

Although it is pertinent to mention that none of the models exhibit the precise pathophysiological events going within the human disease, animal models of MS enable investigation into biological processes that contribute to disease that would not be possible in human subjects, and in some cases, these processes are fairly well preserved between the two species [92]. Thus, animal models of MS are critical in developing an understanding of the pathogenesis of disease, as illustrated by the studies described below.

## Overview of metabolomics studies in EAE model of MS

Given the inherent ethical and safety issues surrounding the use of human patient tissue samples in studying demyelination within the CNS, several animal models of MS have been developed. Of these, EAE is the most widely used preclinical animal model to study inflammation, CNS damage, biomarkers, metabolite interactions, and novel therapeutics [14, 40, 93, 94]. It represents an approximate prototypic disease model that replicates various pathologic features of MS, including peripheral inflammation, a break in the blood–brain barrier, neuroinflammation, axonal loss, gliosis, demyelination, and neurodegeneration [95]. EAE is an antigen-driven inflammatory model commonly induced in mice through immunization against myelin proteins such as myelin oligodendrocyte glycoprotein (MOG), myelin basic protein (MBP), and proteolipid protein (PLP); amongst which C57BL/6 (B6) mice induced with MOG, and SJL mice induced with PLP, represent chronic and RRMS disease courses, respectively [13, 14, 96–98]. Intriguingly, the EAE model also exhibits resolution and remyelination mechanisms that occur in MS, thus making it the most appropriate model for understanding the etiopathogenesis of this autoimmune disease. However, it is pertinent to mention that none of the animal models of MS precisely exhibit the pathophysiological events that take place in the human disease [92]. There are certain differences between established models of EAE that do not entirely mimic MS, such as its clinico-pathology, diverse induction methods, and different treatment response that differs from humans [13]. Despite this, the EAE model has been instrumental in providing new insights into the MS metabolome with the help of rapidly growing metabolomics techniques [99, 100]. Below, we have summarized many of the studies contributing to these insights.

One of the early studies investigating the composition of the metabolome in the context of MS used NMR analysis of the urinary metabolome in MS patients and non-human primates (marmosets) induced with EAE exhibiting MS-like disease manifestations [82]. Although distinct metabolic profiles were observed between monkeys with EAE and non-immunized monkeys, which served as controls, the specific metabolites that were altered could not be identified. Despite this, the study was crucial in combining NMR spectroscopy with pattern recognition to study characteristic metabolite patterns in MS. With time, experimental modifications and innovations in metabolomics approaches have refined our ability to identify alterations in metabolic pathways related to disease, which have opened up new mechanisms to explore the immunopathology and neuropathology driving disease initiation and progression.

To understand the metabolic changes that occur during different stages of EAE development, metabolomics has emerged as a powerful tool in deciphering those changes. To this end, targeted LC–MS and GC–MS were used to investigate the metabolome during the onset and peak of the disease (day 10 and 14 post-immunization, respectively) using CSF obtained from an acute EAE rat model immunized with MBP [101]. LC–MS identified 39 metabolites, while GC–MS identified 64 metabolites, with 18 metabolites identified between these two analytical platforms. Changes in CNS metabolism were reported over the course of the disease as significant differences in the levels of certain metabolites were observed at the onset and peak time points of disease. The altered metabolites included a decrease in arginine, alanine, and branched amino acids at disease onset whereas there was an increase in glutamine, O-phosphoethanolamine, branched-chain amino acids, and putrescine at the peak of disease, with affected pathways associated with processes such as nitric oxide synthesis, excitotoxicity, lipid metabolism, energy metabolism, polyamine synthesis, and antioxidant levels. The findings of the study were consistent with those from other studies using samples from MS patients and other EAE models, although some of the results were controversial to those already published in the literature. Nevertheless, this study was useful in revealing metabolic changes that occur over the course of neuroinflammation in the EAE model.

There is a long-standing need to identify biomarkers that can enable rapid, non-invasive diagnosis, staging, prognosis, and therapeutic interventions in MS [99, 100], and it is preferential that these be identifiable in samples that are easy to attain, such as urine and plasma. In the chronic model of EAE in B6 mice, a distinct metabolic profile was identified in urine during the peak of the disease using the 1H-NMR analytical platform [87]. This study also observed an effect on the metabolome in the urine upon treatment with the MS-drug, fingolimod. In mice induced with EAE that were untreated, altered metabolites included increased levels of fructose and hippurate and decreased levels of urea, oxoglutaric acid, taurine, and citrate compared to healthy and EAE-treated mice. The results of this study may be expanded on to identify possible biomarkers that could be used to monitor treatment responses and provide information about drug efficacy in MS patients. In a similar study, 1H-NMR was used to analyze metabolites in urine and plasma from chronic relapsing EAE (Cr-EAE) at different time points of disease to discriminate between EAE and non-immunized control groups, and to also discern differences in various phases of the disease [102]. Although the metabolic profiles of urine that were identified in this study could not differentiate between different disease states, a distinct plasma profile was observed during clinically silent (day 10, 28) and active (day 14, 38) stages of the disease. The metabolites that changed significantly over time in Cr-EAE compared to control groups, included fatty acids, glucose, and taurine. Collectively, these studies indicate that while urine samples may be useful to distinguish between treated and untreated, it may not be sufficient in being able to detect differences at various stages of the disease. In another study assessing urinary metabolomics, a 2D high-resolution LC–MS/MS technique was applied to the urine of Lewis rats induced with EAE. To study the changes in the urinary metabolome before disease onset when the clinical score was zero, the study looked at three-time points of disease, including before disease onset (day 0), after disease onset (day 7), and at the peak of disease (day 14). This identified alterations in 31 urinary proteins, out of which 17 showed association with various neurological features, with the pathways most impacted including the acute phase response and those associated with metabolic processes. Further, altered expression of 14 proteins that harbor catalytic activity, the disruption of which could cause neuronal damage, were identified. Six out of 7 of the most affected proteins were previously reported to be altered in patient samples. Despite the studies mentioned above, these more recent studies suggest urinary metabolome can be used to identify biomarkers for MS early in the course of the disease.

A notable study by Mangalam et al. utilized an untargeted global metabolomics approach in which ultra-performance liquid chromatography (UPLC)–MS–MS/GC–MS were combined to profile plasma from SJL mice during the chronic phase (day 45) of RR-EAE compared with non-immunized healthy control mice [93]. Out of the 282 metabolites that were detected, 44 were significantly altered. These were involved in lipid, amino acid, nucleotide, and xenobiotic metabolism. Pathway mapping using the Kyoto Encyclopedia of Genes and Genomes (KEGG) database identified 6 major pathways that, when disrupted, were associated with disease severity of EAE, including bile acid biosynthesis, and metabolism of taurine, tryptophan and histidine, linoleic acid, and d-arginine and d-ornithine. Overall, this study showed that these pathways are disrupted in later stage of disease, which suggest their potential to serve as future biomarkers to monitor disease progression in MS and as novel drug targets for metabolic intervention. A recent study from the same group has utilized a global untargeted metabolomics approach using LC–MS/GC–MS to identify the metabolic profile in the urine of B6 mice with chronic EAE (i.e., day 45 post-immunization) [94]. This detected 105 metabolites that were significantly altered, and KEGG assessment showed the most impacted pathway to be phenylalanine–tyrosine pathway. Taken together with the plasma profiling done previously in B6 mice with chronic EAE [40], a total of 8 common metabolites were found to be significantly altered in urine and plasma. Enrichment analysis of these common metabolites showed that the pathways perturbed in both biofluids included phenylalanine metabolism and valine, leucine, and isoleucine biosynthetic pathways. This revealed common metabolic profiles in urine and plasma that could be utilized for non-invasive monitoring of disease progression in MS. However, when compared to previously published data on metabolome of MS patients [78], the only metabolite that was commonly disrupted across studies was creatinine, highlighting the potential limitations of using EAE as a model to study the metabolome in the context of MS [94].

In another recent study, the UHPLC-Orbitrap-MS-based metabolipidomics approach with multivariate analysis was used to study the metabolic changes that occur during disease progression in EAE using plasma samples from mice collected over time as disease progressed from one stage to other (pre-induced, onset, peak, and chronic) [20]. This identified 49 metabolites that were significantly altered in response to EAE pathogenesis, out of which 29 exhibited stage-specific differences. In general, glycerophospholipids and fatty acyls were decreased as disease progressed in mice with EAE relative to controls. In contrast, glycerolipids, taurine-conjugated bile acids, and sphingolipids showed trends of increased levels with disease progression. Collectively, metabolic changes positively correlated with the pathologic status of the disease at each stage, as revealed by increased oxidative stress and inflammation. Therefore, this study demonstrated the need to employ stage-specific treatment of MS due to the metabolic changes that occur as the disease progresses over time.

Despite some of the limitations with using EAE to identify biomarkers in plasma and urine, this model provides a solution for studying MS metabolomics within the CNS, which is otherwise not feasible with human patients. As such, a high-resolution magnetic angle spinning (HRMAS)-based 1H-NMR approach was used to identify the altered metabolites of inflammation during the acute phase of EAE in rats induced with MBP [103]. This uncovered elevated levels of metabolites, including glucose, lactate, ascorbate, and other amino acids associated with energy metabolism, and lower levels of N-acetyl-aspartate (NAA) in the EAE group compared to the control group (non-immunized rats). The positive correlation between lower levels of NAA in the lumbar spinal cord region with clinical signs of the disease was intriguing, which highlights the use of metabolomics to test the therapeutic effects of drugs on EAE/MS.

Some studies of EAE have utilized integrated metabolomics, which has upgraded the accuracy of metabolite detection. Blanchet and colleagues have provided a case report of MBP-induced EAE in Lewis rats where they have used a combination of MS-Orbitrap-NMR metabolomics and proteomics data to refine the separation of metabolites between rats induced with EAE (peripherally inflamed and onset) and healthy control rats [104]. They observed deregulation of energy supply and amino acid metabolism in the CSF of rats induced with EAE that was linked to deregulation of T kininogen 1, lactate or inositol, complement C3, and ceruloplasmin. This study illustrated the utility of a novel fusion approach of proteomics and metabolomics for comprehensive and exhaustive disease profiling with high accuracy of prediction in EAE and MS. Another similar study has also reported the effectiveness of combining NMR metabolomics of plasma and CSF in identifying biomarkers for the onset of neuroinflammation in EAE [105]. It is worth mentioning that the above-discussed metabolomics studies in EAE need further validation in human patient samples due to certain inherent limitations of this model to fully replicate MS.

## Application of metabolomics in MS therapeutics

Metabolomics studies have been instrumental in identifying novel therapeutic targets and small endogenous metabolites with therapeutic potential for MS [17]. The EAE model has been critical for testing the therapeutic potential of these metabolites prior to their use in clinical trials. Indeed, there are multiple studies where potential drugs have been tested in EAE and have proved to be successful in patients including interferon β [106, 107], glatiramer acetate [108, 109], and the anti-VLA-4 antibody [110, 111]. Below, we highlight several of these studies that have shown promising results in using metabolomics to identify biomarkers or targets for therapy to treat MS.

Using an untargeted LC–MS and GC–MS metabolomics approach, Poisson and colleagues reported metabolic alterations in omega-3 and omega-6 metabolism in plasma samples from B6 mice induced with chronic EAE, collected 45 days post-immunization [40]. Out of 324 detected metabolites, 100 of these (mainly lipids) were significantly altered in mice induced with EAE and were linked to mitochondrial function, inflammation, and membrane stability. Bioinformatic analysis revealed that the pathways that were most perturbed were those linked to polyunsaturated fatty acid (PUFA) metabolism, including alpha-linolenic acid (ALA) (omega-3) and linoleic acid (LA) (omega-6) pathways, which is consistent with studies reporting lower levels of PUFA in MS patients and mouse models of the disease [112–116]. Based on findings from the literature, the authors tested the therapeutic potential of resolvin D1 (RvD1), a downstream metabolite of the ALA pathway. Daily oral administration of RvD1 (100 ng per mouse) significantly ameliorated disease progression in B6 mice induced with chronic EAE. This was due to the inhibition of autoreactive T cells and the induction of myeloid-derived suppressor cells (MDSCs), regulatory T cells (Tregs), and monocytes/macrophages and brain microglia with an anti-inflammatory M2 phenotype, all of which inhibit inflammatory responses. Collectively, this provides evidence that metabolites possess drug-like properties and could be a therapeutic option to treat MS. In addition, there have been lipidomics studies based on LC–MS/MS for assessing spectrum of lipid mediators in patient-derived serum and plasma samples. The findings reveal differential regulation and disease severity-dependent synthesis of omega-6 and omega-3-derived lipid mediators, reflecting defects in resolution pathway in MS pathogenesis [117, 118]. The treatment of human leukocytes and brain endothelial cell line hCMEC/D3 with differentially altered pro-resolution mediators including lipoxin A4 (LxA4), lipoxin B4 (LxB4), RvD1, and neuroprotectin D1-modulated inflammatory response by reducing activation of patient-derived monocytes and cytokine production, inhibiting blood–brain barrier dysfunction along with transendothelial migration of monocytes [118]. Altogether, these studies provided mechanistic insights into diagnostic and therapeutic potential of specialized pro-resolving lipid mediators in MS.

The efficacy of metabolomics-based therapeutic intervention in MS to correct metabolic alterations has been tested in cell lines and human patients. One such study by Zhao et al. applied CE-MS technique to map the changes in intracellular metabolites in five different astrocytoma cell lines [119]. Treatment of astrocytes with methionine enkephalin (MENK) resulted in a significant increase in four metabolites, including tyrosine, phenylalanine, methionine, and glycylglycine. Similarly to MENK, glycylglycine inactivated astrocytes, and protected neuron function by inducing expression of remyelination genes, which suggests glycylglycine is the degradation product of MENK. The therapeutic potential of MENK was also tested on a mouse model of MS in which treatment slightly relieved symptoms, resulting in better disease outcomes. Another study used UPLC-MS–MS with multiple analytical techniques in a global untargeted metabolomics approach to evaluate the effect of vitamin D supplementation on the metabolome of plasma samples from a cross-sectional cohort of 27 RRMS patients and 27 healthy subjects that received a 5,000 IU dose of cholecalciferol daily for 90 days [37]. Alterations in metabolites associated with oxidative stress (γ-glutamyl amino acid and glutathione) and xenobiotic metabolism (benzoate and caffeine) were reported in the first cohort of both MS patients and controls, whereas there was a reduction in oxidative stress markers in the healthy subjects that received vitamin D, with this effect being dampened in MS patients. There are certain limitations in this study, including a lack of MS patients with progressive disease, data on the disease-modifying therapies that patients were receiving, last meal taken, and the time of sample collection. In addition, the treatment cohort was focused only on female patients of European descent with low vitamin D levels, which raises concerns over the reproducibility of the data for other MS patients with diverse genetic backgrounds. There is no mention about the impact of genotype on the metabolome after vitamin D supplementation, which seems crucial due to influence of genetic polymorphisms on the vitamin D pathway [120]. Overall, this study suggests genetic factors and gut microbiota may have a strong influence on the metabolome, and that additional validation of these findings in MS patients is warranted before clinical use, particularly in light of the fact that clinical trials to assess the potential of vitamin D in ameliorating MS have been unsuccessful, which brings into question the therapeutic potential of this supplement [121]. Taken together, these studies provide evidence for utilizing metabolomics as a tool to evaluate the efficacy of supplementation for therapeutic intervention in MS.

There are several other studies that have explored the possibility of targeting perturbed metabolic pathways for therapeutic intervention in MS. One of the altered pathways that is consistently involved in tryptophan metabolism is kynurenine pathway, as impaired levels of several metabolites have been linked to higher severity and risk of MS [69, 76, 77, 88]. In addition, a few studies that have used preclinical models of inflammation have reported that metabolites derived from the gut microbiota act as ligands to the aryl hydrocarbon receptor (AHR) present on immune cells and glial cells to induce anti-inflammatory protective effects [122, 123]. Based on these findings, it is plausible that metabolic intervention through supplementation with tryptophan would normalize levels of metabolites to allow activation of AHR and reduce disease severity in MS. Taken together, tryptophan metabolism is emerging as a crucial link to suggest the interaction between the gut microbiome and immune system in the backdrop of immune-mediated diseases.

Recently, the effects of the FDA-approved drug dimethyl fumarate (DMF) on the metabolomic profile of MS patients were assessed relative to immunological changes [124]. Using a global metabolomics approach with LC–MS/GC–MS and weighted correlation network analysis (WGCNA) on the plasma of 18 MS patients collected prior to initiation of DMF and 6 months post-initiation, significant changes in the lipid profile were observed in patients who were treated for 6 months compared to untreated controls. This was accompanied by an increase in phospholipids, lysophospholipids, and plasmalogens, along with a decrease in circulating free fatty acids. In addition, a strong correlation was observed in the altered levels of fatty acids with lymphocyte counts, in particular with the CD8 + T cell subset. Overall, this demonstrated that DMF-induced alterations in lipid metabolism are associated with immunological changes, which highlights the potential to use metabolic markers as a means to monitor therapeutic interventions in MS, allowing for more precise patient-specific treatment. Similarly, another recent study utilized high-resolution 1H-NMR spectroscopy to identify markers for disease activity and therapeutic response by assessing the effect of interferon β 1a therapy on the metabolome of 21 MS patients treated for a varying period of time at 0, 6, 12, and 24 months, compared with 16 healthy controls [125]. Although the sample size was small, making it necessary to repeat this study with a larger dataset, PLS-DA and OPLS-DA models showed differential blood-based metabolite profiles between responders (n = 16) and non-responders (n = 5), based on “no evidence of disease activity” (NEDA-3) derived from absence of relapses, disability progression, and imaging activity. The metabolite distribution showed significant difference between baseline samples and those treated with interferon β therapy, especially for 24 months. The metabolic profile included altered levels of lactate, acetone, 3-hydroxybutyrate, tryptophan, citrate, lysine, and glucose. These findings indicated that tryptophan and energy metabolism are disrupted in patients, and that treatment with interferon β therapy can influence the levels of specific metabolites, suggesting their levels may be useful to monitor as a means to assess the efficacy of treatment.

Intriguingly, the metabolomics approach also provides mechanistic insight into the role of endogenous metabolites in regulating cellular differentiation and programming [126–129]. In particular, the process of oligodendrocyte precursor cell (OPC) differentiation becomes inhibited during progressive stages of MS. Therefore, to identify the endogenous metabolites altered during this process, Beyer et al*.* employed a global untargeted MS-based metabolipidomics approach using hydrophilic interaction chromatography (HILIC) and reversed-phase (RP) LC, combined with liquid chromatography-electrospray ionization-quadruple time-of-flight-mass spectrometry (LC-ESI-QTOF-MS), on cell lysates of in vitro cultured OPCs, which revealed increased levels of taurine during the course of OPC differentiation and maturation [130]. Taking this a step further, the authors exploited the therapeutic potential by treating OPCs with taurine under in vitro conditions. Taurine was effective in maintaining the functionality of oligodendrocytes by inducing OPC differentiation and maturation, potentially by increasing the cellular pool of serine, which acts as a precursor of glycosphingolipid and forms an important component of myelin. Therefore, this study reveals the utility of metabolomics to identify endogenous metabolites that modulate cell differentiation and maturation.

Recently, abnormalities in bile acid metabolism have been described in neurological diseases, which could be attributed to the neuroprotective effects of bile acids by modulating neuroinflammation [131]. In addition, there are reports of abnormalities in bile acids in the blood of MS patients, with altered cholesterol metabolism in astrocytes, associated with neuroinflammation in EAE [93, 132]. Based on these findings, bile acid metabolism may provide new targets for therapeutic intervention in MS. To identify alterations in bile acid metabolism, a recent study by Bhargava et al*.* applied a global untargeted and targeted metabolomics approach using UPLC-MS–MS and LC–MS/MS on plasma samples from multiple cohorts of adult (discovery cohort: 56 RRMS, 51 PMS, and 52 controls; validation cohort: 50 RRMS, 125 PMS, and 75 controls) and pediatric (31 MS and 31 controls) patients [133]. They reported significantly reduced levels of several bile acid metabolites in patients compared to controls, with a more pronounced effect in samples from patients with progressive form of the disease. They went on to demonstrate that receptors involved in bile acid signaling, including farnesoid X receptor (FXR) and G-protein-coupled bile acid receptor (GPBAR1), were expressed on glial and immune cells obtained from white matter lesions in MS brain tissue. Further, they investigated the therapeutic implications of bile acid on cellular and animal models of MS using the secondary bile acid, tauroursodeoxycholic acid (TUDCA). TUDCA showed dose-dependent anti-inflammatory effects on astrocytes and microglia under in vitro conditions by inhibiting astrocyte and microglia polarization into neurotoxic and pro-inflammatory phenotypes, respectively. Supplementing EAE B6 mice with TUDCA ameliorated disease progression by reducing neuroinflammation, mediated through GPBAR1 present on astrocytes and macrophages. Based on these promising findings, there is an ongoing clinical trial (NCT03423121) to evaluate the effects of TUDCA supplementation on the circulating metabolome, gut microbiota, and peripheral immune response. Overall, this study potentiates the importance of metabolomics in identifying the altered metabolic pathways associated with MS.

**Fig. 3:**
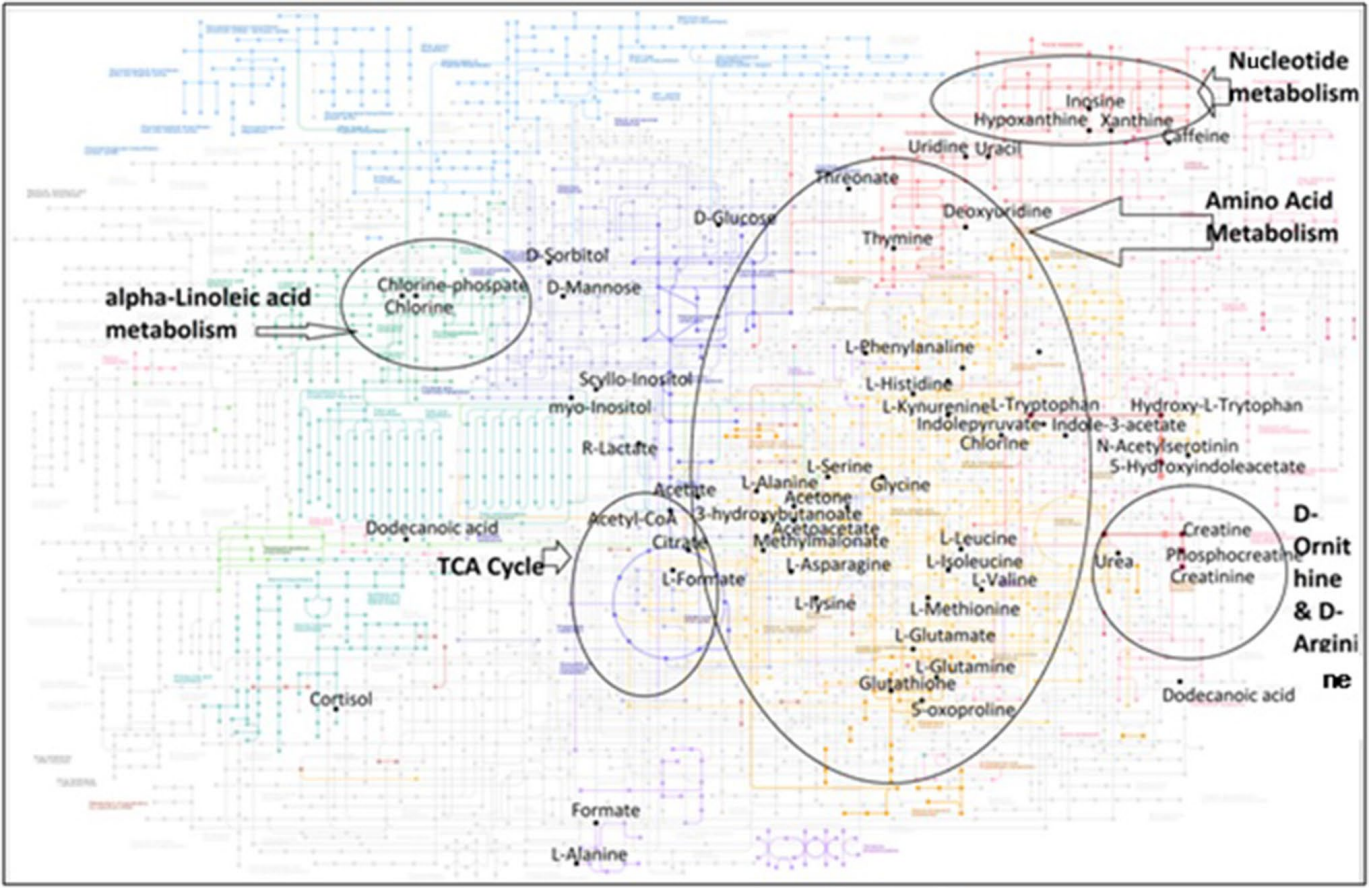
KEGG-based map of altered metabolites and associated pathways derived from the major metabolomics studies in MS patients and EAE

Collectively, the findings outlined above demonstrate the potential for metabolic intervention as an alternate treatment approach to correct endogenous metabolic abnormalities in MS. Although the etiology behind metabolic abnormalities in MS remains unknown, there are a number of ongoing studies assessing the precise effects of altered metabolites on disease outcomes. Many of the studies described above utilizing the samples from MS patients and EAE model (described in Tables 1 and 2) provide important insights that lay the groundwork for future studies to further understand the role of the metabolome in contributing to MS pathogenesis, and its use as a predictive and therapeutic modality for this disease. To bring the outcome from different studies into perspective, metabolites from human MS studies mapped to KEGG IDs based on metaboanalyst ID conversion module were overlaid on KEGG (hsa) pathways to highlight metabolites or pathways in which MS-related metabolites were enriched (https://www.genome.jp/kegg/) [134]. Similarly in EAE mouse data, we observed overlap of some of these metabolites such as d-glucose, citrate, taurine, linoleate, urea, l-leucine, and octadecatrienoic acid. Figure 3 provides the pictorial representation of altered metabolites and associated pathways using KEGG analysis and metaboanalyst for the major metabolomics studies in MS patients and EAE model.

**Table 1:** Comparison between major metabolomics studies in MS patient samples^#^

Study	Approach/platform	Sample size	Sample type	Key metabolites/pathways detected
Bruhn et al. 1992 [38]	MRS with MRI	8 pediatric MS, 10 pediatric controls	In vivo white matter	Choline, myo-inositol, N-acetyl-aspartate, creatine
Lynch et al. 1993 [45]	NMR	30 PMS, 27 controls	CSF	Acetate, formate
Nicoli et al. 1996 [46]	MRS	19 MS, 17 controls	CSF	Lactate, fructose, creatinine, phenylalanine
Simone et al. 1996 [47]	MRS	52 MS, 32 non-MS, 18 controls	CSF	Formate, lactate
‘t Hart et al. 2003 [82]	NMR	10 MS, 11 controls, 20 non-MS	Urine	Aspartic acid, inositol, taurine
Lutz et al. 2007 [39]	MRS	21 active MS, 12 inactive MS	CSF	BHIB, lactate
Regenold et al. 2008 [49]	GC–MS	31 RR, 54 SP, 18 controls	CSF	Sorbitol, fructose, lactate
Gonzalo et al. 2012 [51]	LC–MS/UHPLC–MS	9 MS, 9 non-MS	CSF	8-iso-prostaglandin F2α
Mehrpour et al. 2013 [67]	NMR	23 MS, 28 controls	Serum	Glucose, valine
Vingara et al. 2013 [90]	MRS with MRI	27 RRMS, 14 controls	In vivo white matter	N-acetyl-aspartate, choline, N-acetyl aspartyl glutamate, glutamine, glutamate, scyllo-inositol, creatine, phosphocreatine, lipids
Pruss et al. 2013 [117]	LC–MS/MS	20 RRMS	Serum CSF	15-Hydroxyeicosatetraenoic acids, prostaglandin E2, resolvin D1, neuroprotectin D1, lipoxin A4
Dickens et al. 2014 [78]	NMR	Cohort A: 22 RRMS, 46 SPMS, 17 PPMS, 14 controls Cohort B and C: 13 RRMS, 21 SPMS, 18 controls	Serum	Fatty acids, phosphocholine, N-acetyl species, glucose, lactate
Reinke et al. 2014 [41]	NMR	15 MS, 17 non-MS	CSF	Mannose, choline, myo-inositol, threonate, citrate, mannose, phenylalanine, 3-hydroxybutyrate, 2-hydroxyisovalerate
Moussallieh et al. 2014 [73]	NMR	44 NMO, 47 MS, 42 controls	Serum	Scyllo-inositol, glutamine, acetate, glutamate, lactate, lysine
Pieragostino et al. 2015 [52]	MALDI-TOF–MS LC–MS/MS	13 RRMS, 12 non-MS	CSF	Phospholipid metabolism
Cocco et al. 2016 [69]	NMR	73 MS, 88 controls	Plasma	Glucose, 5-OH-tryptophan, tryptophan, increased 3-OH-butyrate, acetoacetate, acetone, alanine, choline
Gebregiworgis et al. 2016 [87]	NMR	8 RRMS, 9 NMSOD, 7 controls	Urine	Creatinine, 3-hydroxybutyrate, 3-hydroxyisovalerate, methylmalonate
Villoslada et al. 2017 [70]	UHPLC–MS	Cohort A: 238 MS, 74 controls Cohort B: 61 MS, 41 controls	Serum	Hydrocortisone, glutamic acid, tryptophan, eicosapentaenoic acid, 13S-hydroxyoctadecadienoic acid, lysophosphatidylcholines, lysophosphatidylethanolamines
Kim et al. 2017 [53]	NMR	50 MS, 57 NMOSD, 17 controls	CSF	2-Hydroxybutyrate, acetone, formate, pyroglutamate, acetate, glucose, citrate, lactate, isoleucine, valine
Jurynczyk et al. 2017 [74]	NMR	34 RRMS, 54 AQP4-Ab NMOSD, 20 MOG-Ab	Plasma	Low-density lipoproteins, high-density lipoproteins, histidine, glucose, lactate, alanine, formate, leucine, low myo-inositol
Lazzarino et al. 2017 [75]	HPLC	518 MS, 167 controls	Serum	Hypoxanthine, xanthine, uric acid, inosine, uracil, β-pseudouridine, uridine, creatinine, lactate
Lim et al. 2017 [76]	UHPLC GC–MS	Cohort A: 50 RRMS, 20 SPMS, 17 PPMS, 49 controls Cohort B: 44 RRMS, 15 SPMS Cohort C: 9 RRMS, 20 SPMS, 6 controls	Serum	Kynurenic acid, quinolinic acid, kynurenine, tryptophan
Bhargava et al. 2017 [37]	UPLC–MS–MS	27 RRMS, 27 controls	Plasma	γ-Glutamyl amino acid, glutathione, benzoate, caffeine
Bhargava et al. 2019 [124]	LC–MS/GC–MS	18 MS, 18 controls	Plasma	Phospholipids, lysophospholipids, plasmalogens
Herman et al. 2018 [54]	ELISA, LC–MS	30 RRMS, 16 SPMS, 10 controls	CSF	20β-Dihydrocortisol, indolepyruvate, 5,6-dihydroxyprostaglandin F1a
Nourbakhsh et al. 2018 [77]	LC/GC–MS	Untargeted group: 66 pediatric and CIS, 66 pediatric controls Targeted discovery group: 82 pediatric MS, 50 pediatric controls Validation group: 92 pediatric MS, 50 pediatric controls	Serum	Tryptophan, indole lactate, kynurenine
Stoessel et al. 2018 [79]	LC–MS	33 PPMS, 10 RRMS, 63 controls, 40 Parkinson’s disease	Plasma	Glycerophospholipids, linoleic acid, lysoPC
Herman et al. 2019 [55]	HRMS	30 RRMS, 16 SPMS, 10 controls	CSF	Kynurenate, 5-hydroxytryptophan, 5-hydroxyindoleacetate, N-acetylserotonin, indole-3-acetate, thymine, glutamine, uridine, deoxyuridine
Andersen et al. 2019 [18]	2D GCxGC-TOFMS	12 MS, 13 controls	Serum	Pyroglutamate, laurate, acylcarnitine C14:1, N-methylmaleimide, phosphatidylcholines
Cicalini et al. 2019 [91]	LC–MS/MS	12 MS, 21 controls 12 MS, 10 controls	Tears Serum	Phospholipids (sphingomyelins), amino acids, acylcarnitines
Lorefice et al. 2019 [125]	NMR	21 MS; 16 controls	Blood	Lactate, acetone, 3-OH-butyrate, tryptophan, citrate, lysine, glucose levels
Kasakin et al. 2019 [71]	LC–MS/MS	22 RRMS, 22 controls	Plasma	Glutamate, leucine-isoleucine, valine, decenoylcarnitine
Nogueras et al. 2019 [56]	LC–MS GC–MS	53 MS, 54 non-MS	CSF	Glycerolipids, sterol lipids, fatty acids arachidic acid (18:3n3 and 20:0), glycerophospholipids, sphingolipids,
Podlecka-Piętowska et al. 2019 [57]	NMR	19 MS, 19 controls	CSF	Acetone, choline, urea, 1,3-dimethylurate, creatinine, isoleucine, myo-inositol, leucine, 3-OH butyrate, acetyl-CoA
Kooij et al. 2019 [118]	LC–MS-MS	26 RRMS, 12 PMS, 15 controls	Plasma	Lipoxin A4, lipoxin B4, resolvin D1, protectin D1
Bhargava et al. 2020 [133]	UPLC–MS–MS	Adult discovery cohort: 56 RRMS, 51 PMS, 52 controls Adult validation cohort: 50 RRMS, 125 PMS, 75 controls Pediatric cohort: 31 MS, 31 controls	Plasma	Bile acid
Carlsson et al. 2020 [58]	LC-HRMS FIA-HRMS	12 SPMS, 12 controls	CSF	Glycine, asymmetric dimethylarginine, glycerophospholipid PC-O (34:0), hexoses
Murgia et al. 2020 [59]	NMR, LC–MS/GC–MS	22 RRMS, 12 PPMS	CSF Serum	Lipids, biogenic amines, amino acids
Sylvestre et al. 2020 [72]	NMR	28 RRMS, 18 controls	Plasma	Arginine, isoleucine, citrate, serine, phenylalanine, methionine, asparagine, histidine, myo-inositol
Gaetani et al. 2020 [88]	HPLC–MS/MS	47 RRMS, 43 controls	Urine	Tryptophan, kynurenine, anthranilate, indole-3-propionic acid

^#^Non-MS: other neurological diseases; controls: healthy subjects or patients without neurological diseases

2D 2 dimensional; AQP4-Ab aquaporin-4 antibody; CIS clinically isolated syndrome; CSF cerebrospinal fluid; ELISA enzyme-linked immunosorbent assay; FIA flow injection analysis; GC–MS gas chromatography–mass spectrometry; HPLC high-performance liquid chromatography; HRMS high-resolution mass spectrometry; LC–MS liquid chromatography–mass spectrometry; MALDI matrix-assisted laser desorption ionization; MOG-Ab myelin oligodendrocyte antibody; MRI magnetic resonance imaging; MRS magnetic resonance spectroscopy; MS multiple sclerosis; NMO neuromyelitis optica; NMOSD neuromyelitis optica spectrum disorder; NMR nuclear magnetic resonance; PMS progressive multiple sclerosis; PPMS primary progressive multiple sclerosis; RRMS relapsing–remitting multiple sclerosis; SPMS secondary progressive multiple sclerosis; TOF time of flight; UHPLC ultra high-pressure liquid chromatography; UPLC ultra-performance liquid chromatography

**Table 2:** Comparison between the main metabolomics studies in cellular models and EAE

Study	Approach/platform	Model	Matrix	Key metabolites/pathways detected
‘t Hart et al. 2003 [82]	NMR	EAE; marmoset monkey	Urine	Aspartic acid, inositol, taurine
Blanchet et al. 2011 [104]	MS-Orbitrap-NMR Proteomics	EAE; Lewis rat	CSF	T kininogen 1, lactate or inositol, complement C3, ceruloplasmin
Noga et al. 2012 [101]	LC–MS/GC–MS	EAE; Lewis rat	CSF	Arginine, alanine, branched amino acids, glutamine, putrescine, O-phosphoethanolamine
Mangalam et al. 2013 [93]	UPLC–MS–MS/GC–MS	RR-EAE; SJL mice	Plasma	Bile acid biosynthesis, taurine metabolism, tryptophan and histidine metabolism, linoleic acid, d-arginine and d-ornithine metabolism
Gebregiworgis et al. 2013 [135]	NMR	Chronic EAE; B6 mice	Urine	Fructose, hippurate, urea, oxoglutaric acid, taurine, citrate
Dickens et al. 2015 [102]	NMR	Chronic-relapsing EAE; Biozzi ABH mice	Urine Plasma	Fatty acids, glucose, taurine
Zhao et al. 2015 [119]	CE-MS	Astrocytoma cell lines	Astrocytes	Tyrosine, phenylalanine, methionine, glycylglycine
Poisson et al. 2015 [40]	LC–MS/GC–MS	Chronic EAE; B6 mice	Plasma	Alpha-linolenic acid, linoleic acid
Zhao et al. 2017 [136]	2D LC–MS/MS	EAE; Lewis rat	Urine	Enzymes, peptidases
Battini et al. 2018 [103]	HRMAS-NMR	Acute EAE; Lewis rats	CNS tissue	Glucose, lactate, ascorbate, amino acids, N-acetyl-aspartate
Beyer et al. 2018 [130]	HILIC, RPLC, LC-ESI-QTOF-MS	In vitro OPC culture	Cell lysate	Taurine
Singh et al. 2019 [94]	LC–MS/GC–MS	Chronic EAE; B6 mice	Urine Plasma	Phenylalanine metabolism and valine, leucine, isoleucine metabolic pathway
Lee et al. 2019 [20]	UHPLC-Orbitrap-MS	EAE; B6 mice	Plasma	Glycerophospholipids, fatty acyls glycerolipids, taurine-conjugated bile acids, sphingolipids

2D 2 dimensional; CE capillary electrophoresis; CSF cerebrospinal fluid; EAE experimental autoimmune encephalitis; GC–MS gas chromatography–mass spectrometry; HILIC hydrophilic interaction chromatography; HRMAS high-resolution magnetic angle spinning; LC–MS liquid chromatography–mass spectrometry; LC-ESI-QTOF-MS liquid chromatography-electrospray ionization-quadruple time-of-flight-mass spectrometry; MS mass spectrometry; NMR nuclear magnetic resonance; OPC oligodendrocyte progenitor cells; RPLC reversed-phase liquid chromatography; RR relapsing–remitting; UHPLC ultra high-pressure liquid chromatography

## Conclusions and perspectives

There is a pressing need for reliable disease-specific diagnostic and prognostic biomarkers that would allow us to understand the precise patho-etiological mechanisms of MS, which will enable the development of more precise and personalized medicine. Metabolomics-based studies have provided useful insights into the specific metabolic pathways disrupted in MS such as amino acid, lipid, and energy metabolism, making them a promising tool to better understand the disease. Evidence suggests that metabolite profiles can serve as a potential signature to diagnose MS, stage the disease course, predict its progression, and assess the effects of drug responses. Owing to this, there has been a significant increase in metabolic assays to study the initiation and progression of MS. Altogether, there has been a certain level of overlap in the metabolite profiles identified in various studies so far, yet a reproducible MS-specific metabolome-based signature remains to be identified. This is likely attributed to the variability in experimental conditions and techniques being used for metabolic profiling, which includes disease heterogeneity, a lack of stringent inclusion criteria, batch effects due to diverse sample type, variable sample size, heterogeneity in study populations, and, sample handling, sample preparation, and data analysis methods. To enhance the quality of metabolomics studies, these factors need to be taken into account while designing the study and comparing outcome across studies before making any conclusion.

The misdiagnosis of MS is a persistent problem as there is no definitive test, which poses a considerable clinical and psychosocial burden for patients and medical workers [23]. This accentuates the urgent need for MS-specific biomarkers with diagnostic, prognostic, and therapeutic potential. This would also minimize the chances of misdiagnosis to a large extent as MS can be easily differentiated from non-MS mimicking brain diseases with the help of a differential diagnosis. While the CSF presents the most reliable biological matrix for MS studies, its collection is tricky due to the invasive nature of spinal tap procedure and poses certain limitations [62]. In contrast to CSF, blood-based and urine biomarkers can be obtained safely and rapidly for diagnostic and prognostic purposes with no side effects. So far, the most reliable blood-based biomarkers have been antibodies against natalizumab and interferon β drugs through which drug efficacy and related side effects are assessed, making them clinically useful [137–139]. In addition to these biomarkers, metabolites offer an attractive source of biomarkers given their dynamic levels in response to changes in metabolism during health and disease state. However, one of the limitations of metabolomics studies in the EAE model is the discrepancy between their relevance to human patients, which necessitates the findings in animal models to be validated and confirmed in patients through clinical data. The lack of consensus across studies warrants an integrated approach that connects metabolomics to other omics data and that obtained from analysis of the gut microbiota, as well as clinical, imaging, and laboratory reports [104, 140]. The gut microbiome has been shown to impact metabolic profiles, therefore, combining metabolomic studies with data from the microbiota may provide further leads into the effects on the global metabolome in MS [141, 142]. Bioactive metabolites can be identified and would undoubtedly provide an in-depth understanding of disruptions that are taking place within an individual with underlying pathology [143].

Detecting metabolic networks that can be manipulated to generate novel therapeutic targets will help to facilitate precision medicine. Although metabolomics is a promising area of investigation, certain limitations prevent the application of many of the findings that arise from these studies. The current need is to advance our capabilities to diagnose MS, which would facilitate early initiation of appropriate treatment in patients and likely slow down disease progression and the consequent disability. In addition, being able to detect changes in the metabolome may be beneficial in examining the impact of pharmacological and non-pharmacological therapies used in clinical trials of MS. However, the translation of metabolomics studies to the clinic will require upgrading analytical platforms, instrumentation, extraction and recovery methods, and data analysis. In addition, consistency in study design should be considered, including study size and effect of confounding factors such as diet and medications. Finally, the integration of targeted and untargeted metabolomics approaches should be applied as this would increase the spectrum of metabolite detection, allowing for expansive and comprehensive metabolomic profiling in MS. Thus, while promising, the metabolomics approach needs further refinement to generate meaningful data that can be translated to the clinic, and the current focus should be on using multiple platforms that would generate more stage-specific reproducible data with the ultimate goal of improving disease management in MS patients.
